# Influence of Severe Plastic Deformation and Aging on Low Cycle Fatigue Behavior of Al-Mg-Si Alloys

**DOI:** 10.3390/ma17092148

**Published:** 2024-05-03

**Authors:** Wonhoe Kim, Kibeom Kim, Kwonhoo Kim

**Affiliations:** 1 Department of Material, Korea Polytechnic, 51-88 Oedongballim-ro, Seongsan-gu, Changwon 51518, Republic of Korea; peace0211@gmail.com; 2 Department of Advanced Materials Science, The University of Tokyo, 5-1-5 Kashiwanoha, Kashiwa 272-8561, Japan; k.kim23a@ams.k.u-tokyo.ac.jp; 3 Department of Metallurgical Engineering, Pukyong National University, 45 Yongso-ro, Nam-gu, Busan 48513, Republic of Korea

**Keywords:** ultrafine-grained Al-Mg-Si alloy, precipitation hardening, low cycle fatigue, cyclic softening, microstructural stability

## Abstract

Strain-controlled low cycle fatigue (LCF) tests were conducted on conventionally grained (CG) and ultrafine-grained (UFG) Al-Mg-Si alloys treated under various aging conditions. In the cyclic stress response (CSR) curves, CG peak-aged (PA) alloys showed initial cyclic hardening and subsequent saturation, whereas CG over-aged (OA) alloys displayed cyclic softening behavior close to saturation. The UFG materials exhibited continuous cyclic softening except for UFG 3; it originates from the microstructural stability of the UFG materials processed by severe plastic deformation (SPD). Using a strain-based criterion, the LCF behavior and life of the CG and UFG materials were analyzed and evaluated; the results are discussed in terms of strengthening mechanisms and microstructural evolution. In the CG materials, the LCF life changed markedly owing to differences in deformation inhomogeneity depending on the precipitate state. However, the UFG materials displayed a decreasing LCF life as cyclic softening induced by dynamic recovery became more severe; additionally, a relationship between the microstructural stability of the UFG materials and the cyclic strain hardening exponent *n′* was suggested.

## 1. Introduction

As part of efforts to achieve carbon neutrality and environmental protection, there is increasing demand for reducing the weight of vehicles and other major means of transportation. The most efficient way to reduce weight is to replace current commonly used metallic materials with lightweight metals such as an Al alloy [1,2]. An Al-Mg-Si alloy has excellent specific strength, formability, extrudability, and surface treatment properties compared with those of other Al alloys, and the demand is continuously increasing in industrial fields [3,4,5,6].

In an Al-Mg-Si alloy, precipitation hardening and grain refinement are used as strengthening mechanisms; however, various attempts have been made to control the microstructure to overcome the limitations of relatively low strength compared with that of major structural materials such as steel [7,8,9]. One of the most effective methods that can be applied to metallic materials to maximize strengthening mechanisms, for example, by a uniform improvement in strength and toughness, is to make ultrafine-grained (UFG) and nanocrystalline (NC) materials via severe plastic deformation (SPD); various processes such as equal channel angular pressing (ECAP) have been applied [5,10,11] to this end.

Al-Mg-Si alloy is a precipitation hardening alloy; therefore, a fundamental and comprehensive understanding of precipitation hardening is required to effectively improve the mechanical properties of the alloy. For precipitation hardening, the effect of the strengthening mechanism may vary owing to static precipitation caused by heat treatment and dynamic precipitation caused by plastic deformation, among other factors. More research has been conducted on static precipitation [12,13]. Less research has focused on the effects of dynamic precipitation on the mechanical properties of the alloys [14,15,16]; especially dynamic precipitation that occurs during SPD and composite precipitation that occurs via subsequent heat treatment after SPD.

Fatigue testing is widely recognized as an effective method for evaluating the reliability of structural materials, leading to numerous studies on Al alloys [17]. Previous research has demonstrated that precipitation-hardening Al alloys, such as Al-Cu [18,19], Al-Mg-Si [20,21,22], and Al-Zn alloys [23,24], undergo microstructural changes during cyclic deformation [25]. The preceding discussions outlined the state-of-the-art in the cyclic deformation response of precipitation-hardening Al alloys, highlighting their inherent nature concerning dislocation–precipitate interactions [22]. Nandy et al. [22] reported that the cyclic deformation responses are significantly influenced by the aging state of Al alloys. Due to the variation in precipitate states among Al alloys, generalizing the cyclic deformation response and specific dominant mechanisms for dislocation–precipitate interactions without detailed experimental investigations is challenging [22]. While strain-controlled low cycle fatigue (LCF) tests have been extensively studied for general Al alloys, few studies have focused on precipitation-hardening UFG and NC alloys using various heat treatments. Hence, additional research is essential [26]. Recently, several efforts have been made to enhance the LCF properties of UFG alloys through microstructure optimization [27,28]. An et al. [28] reported that the deterioration of LCF properties in UFG alloys is primarily attributed to cyclic softening caused by microstructural instability.

Analyzing LCF behavior is crucial for designing safe and efficient materials and for determining their suitable applications in structural elements subjected to cyclic loading. To date, the evaluation of and relationship between plastic strain amplitude ($\varepsilon_{pa}$) and LCF life have been performed mainly based on strain-based methodologies, especially using the power law relationship proposed by Coffin and Manson [29,30]. The phenomenon of cumulative damage in alloys under cyclic deformation results from a combination of high and low cycle fatigue regimes. Therefore, it can be estimated by integrating the Basquin model [31].

In this study, Al-Mg-Si alloys manufactured under various process conditions were selected, including conventionally grained (CG) alloys under aging conditions and UFG alloys with and without post-SPD aging treatment. Furthermore, the LCF behavior and properties of the alloys were evaluated and analyzed using strain-based methods, and damage mechanisms were discussed in terms of evolution of the microstructure.

## 2. Materials and Methods

Commercial 6005 Al extruded alloy was used as the base material for this investigation; the chemical composition of the 6005 Al alloy is shown in Table 1. All materials used in this study were maintained at 530 °C for 90 min and then quenched in water for solid solution (SS) before subsequent aging or the SPD process. After quenching, the CG group of materials was aged at 175 °C for 8 h to achieve peak aging (CG PA) or aged at 175 °C for 8 d to achieve over-aging (CG OA). ECAP was selected as the SPD process to achieve the UFG condition; note that all ECAP processes used in this study were in the 4 pass state. ECAP was performed via route Bc using a solid die with Φ = 90°, Ψ = 40°, and a speed of 3 mms^−1^ at 150 °C. For some materials in the UFG group, ECAP was performed after SS treatment to assess the changing properties after SPD under supersaturated conditions (UFG 1). To compare the characteristics that change when applying the peak-aged (PA) conditions of CG materials after SPD, some of the materials of the UFG group were aged at 175 °C for 8 h after ECAP (UFG 2). As the maximum tensile strength was observed during low-temperature aging at 100 °C for 25 h after ECAP, the other materials of the UFG group were aged at 100 °C for 25 h after ECAP (UFG 3).

Tensile tests were conducted using an Instron servo-hydraulic testing machine under a constant strain rate of 1×10^−3^ s^−1^ at room temperature (RT); the specimen for the tensile test was made in accordance with ASTM B 557M-06 [32].

LCF tests were performed under total strain control (strain ratio R_ε_ = −1) in a computerized Instron testing system. A triangular waveform initiated from tension to compression with total strain amplitude ($\varepsilon_{ta}$) ranging 0.6%–1.2% was applied at RT under a constant strain rate of 3×10^−3^ s^−1^. The specimen for the LCF test was made according to ASTM E 606-04 [33]; each LCF test was continued until the failure of the specimen, which means complete separation of the specimen.

The microstructural characterization of the processed samples was performed using electron back scattered diffraction (EBSD, S4300, Hitachi, Japan) and transmission electron microscopy (TEM, JEM-2000, JEOL, Japan, and Tecnai F20 G2, FEI, USA), respectively. The EBSD samples were prepared by mechanical polishing followed by cloth polishing; furthermore, electrochemical polishing was performed in a 5% perchloric acid solution in methanol in a DC power supplier at 10 V, −20 °C. The TEM samples were prepared by reducing the samples to 0.1 mm via mechanical polishing followed by punching; a 3 mm diameter punch was used to make a circular disk of 0.1 mm thickness. Twin jet polishing of the samples was subsequently performed using an etchant of 25% nitric acid and 75% methanol at −20 °C and 25 V for the TEM observation. The TEM examinations were performed, before and after the LCF tests, at an acceleration voltage of 200 kV.

## 3. Results and Discussion

### 3.1. Microstructure and Monotonic Tensile Properties

**Figure 1:**
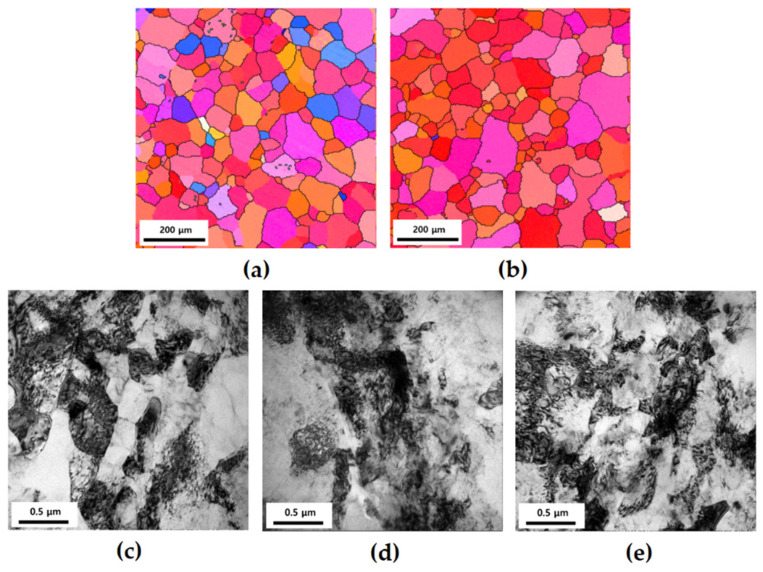
Electron back scattered diffraction and transmission electron microscopy images of 6005 Al alloy before cyclic deformation: (**a**) CG PA; (**b**) CG OA; (**c**) UFG 1; (**d**) UFG 2; (**e**) UFG 3.

Figure 1 shows the EBSD and TEM bright field images of the 6005 Al alloy processed under various conditions. The results of the EBSD analysis indicate that the grain size of CG PA and CG OA is 120 μm and 128 μm, respectively (Figure 1a,b); hence, it is believed that 175 °C is not sufficiently high for apparent grain growth to be observed. The TEM images of the UFG group are shown in Figure 1c–e. For UFG 1, many dislocations are visible inside the grains; the grain size is between 400 and 800 nm, which is similar to that used in previous research (Figure 1c) [26]. The dislocation density of UFG 2 appears to be marginally reduced and the grain size is slightly bigger than that before aging (Figure 1d). The TEM micrograph of UFG 3 indicates that the size of the grain does not change markedly and many dislocations are visible inside the grain (Figure 1e).

Table 2 lists the monotonic tensile properties of the 6005 Al alloy processed under the various conditions used in the study. For CG OA, both strength and elongation decrease compared with those for CG PA. A decrease in elongation has been reported to be due to over-aging and the presence of excess Si [34,35]. Thus, it can be confirmed that the ductility characteristics of the 6005 Al alloy react sensitively depending on the aging conditions. Because UFG 2 is a material to which the peak aging of the CG material has been applied after SPD, and both strength and elongation decrease due to over-aging, resulting in the lowest tensile toughness.

Tensile toughness and the TS/YS ratio have the potential to predict LCF behavior because they depend on the combination of strengthening mechanisms a material can accommodate [36]. For CG materials without precipitation hardening, tensile toughness can increase mainly owing to grain refinement; however, precipitation hardening materials can be markedly affected by precipitation conditions, which can lead to varying results [37]. For materials processed by SPD, those with equiaxed UFG (4 pass condition) had higher tensile toughness compared with that of materials with anisotropy (1 pass condition) [37,38]. The TS/YS ratio can vary depending on the combination of strengthening mechanisms but generally tends to decrease in proportion to the extent of strengthening mechanisms applied. Therefore, the TS/YS ratio can be used as a parameter to determine the degree of strengthening mechanism that can be accommodated by the material, and may be used to predict the behavior of the material when additional deformation occurs. For UFG 1, the TS/YS ratio is the lowest because of the various strengthening mechanisms applied during SPD; therefore, the TS/YS ratio may be increased using appropriate heat treatment after SPD; and optimal tensile properties may be obtained by accomplishing a balance between ductility and strength. Various efforts have been made to overcome the shortcomings of UFG materials processed by SPD and improve their monotonic tensile properties through heat treatment such as low-temperature annealing [39]; the UFG 3 fabricated in this study can be a good candidate for such processes.

### 3.2. Low Cycle Fatigue Behavior

#### 3.2.1. Cyclic Stress Response

**Figure 2:**
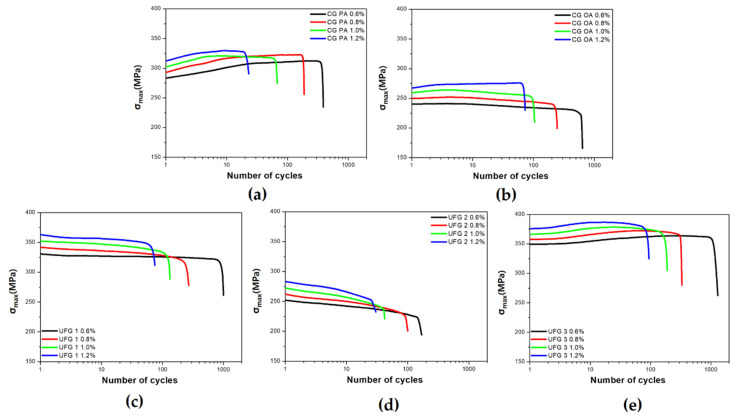
Stress amplitude evolution with number of cycles at different total strain amplitudes ($\varepsilon_{ta}$): (**a**) CG PA; (**b**) CG OA; (**c**) UFG 1; (**d**) UFG 2; (**e**) UFG 3.

The cyclic stress response (CSR) curves of the 6005 Al alloy under various conditions are presented in Figure 2. The CSR curves of CG PA indicate that initial cyclic hardening and subsequent saturation occur at $\varepsilon_{ta}$ ranging between 0.6 to 1.2% (Figure 2a). The initial cyclic hardening may occur because the dislocations are unable to shear the PA precipitates (β″) easily. Thus, the dislocation density may increase with increasing number of cycles during LCF. The initial hardening of CG PA is similar to the results obtained in previous research [40,41,42]. The saturation of the CSR curves after initial hardening can be explained by the fact that the precipitates, which initially prevent the movement of the dislocations, are eventually sheared by the repeated to-and-fro motions of dislocations [41]. Therefore, the softening effects resulting from the shearing of the precipitates and the hardening effects resulting from the increased dislocation density appear to balance each other, resulting in saturation behavior.

In CG OA, the movement of the dislocation could not be limited owing to loss of coherency of β, the main strengthening precipitate, and its slip irreversibility is lower [43] than that of the other conditions; a softening behavior is displayed close to saturation (Figure 2b), [42,43,44]. The tendency for the slope of CSR to change as $\varepsilon_{ta}$ increases is similar to that observed in previous studies [20,45]. In CG OA, the influence of the non-shearable precipitates on the slip behavior is crucial; the LCF behavior and life due to the effects of precipitates will be discussed in the later sections.

The cyclic softening observed for UFG 1 and 2 (Figure 2c,d) is similar to the results obtained in previous studies in which SPD-treated UFG materials displayed cyclic softening [36]. In many UFG materials, softening tends to occur predominantly; this phenomenon is consistent with the softening behavior that materials with a high degree of work hardening exhibit during LCF and is known to be recovery by rearrangement of dislocations [46]. Various mechanisms account for cyclic softening, including dynamic recovery, dynamic recrystallization, grain coarsening, and the formation of shear bands [28,47]. For UFG and NC materials, the LCF behavior and life decrease owing to cyclic softening [28,47]; hence, suppressing this behavior could improve LCF properties. The TEM micrographs in Figure 8d,e indicate that dynamic recovery occurs after LCF testing of UFG 1 and 2; none of the other phenomena mentioned above are observed. Dynamic recovery may be suppressed during LCF by the combination and effective application of strengthening mechanisms, therefore reducing the dislocation mean free path [48] via LCF could assist in suppressing dynamic recovery. Hence, identifying the strengthening mechanisms applicable to each UFG material would assist in understanding the phenomenon. The faster cyclic softening of UFG 2 than that of UFG 1 may occur because the main factor that can contribute to the strengthening of UFG 2 is grain refinement, which is due to the relatively low dislocation density and reduced precipitation hardening effect caused by over-aged (OA) precipitates. By contrast, UFG 1 undergoes grain refinement and has a high dislocation density and can be expected to exhibit more effective precipitation hardening due to dynamic precipitation occurring in the SPD and LCF processes [18,49]. Thus, the rapid cyclic softening displayed by UFG 2 compared with that of UFG 1 could stem from the limited strengthening mechanisms present in the alloy. UFG 3 initially exhibits weak cyclic hardening followed by slight softening close to saturation, in contrast to the continuous cyclic softening observed in the other UFGs. UFG 3 displays strengthening mechanism characteristics such as precipitation hardening, grain refinement, and high dislocation density, and is a material in which static and dynamic precipitates effectively strengthen the materials via low-temperature peak-aging after SPD. In addition, the tendency of softening can be reduced by increasing TS/YS through low-temperature aging after SPD. A major factor in softening is the rearrangement of dislocations, which can proceed actively when the mean free path of dislocation increases; however, depending on the combination of strengthening mechanisms displayed by UFG 3, continuous softening can be suppressed most effectively by impeding the movement of dislocations.

**Figure 3:**
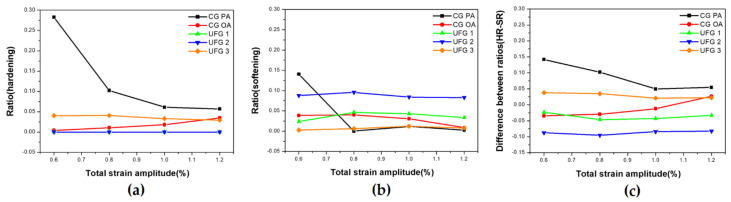
Variations of values of cyclic hardening ratio and softening ratio with total strain amplitude (*ε_ta_*): (**a**) ratio (hardening); (**b**) ratio (softening); (**c**) difference between ratios (HR-SR).

Figure 3 shows the results of an evaluation using the hardening ratio (HR) and softening ratio (SR) Equations (1) and (2) [45,50,51] below to understand hardening and softening behavior more effectively during LCF.

HR = (Δ*σ_max_* − Δ*σ_first_*)/Δ*σ_first_*, (1)

SR = (Δ*σ_max_* − Δ*σ_half_*)/Δ*σ_max_*, (2)

where Δ*σ_max_*, Δ*σ_first_*, and Δ*σ_half_* represent the maximum stress amplitude, stress amplitude at the first cycle, and stress amplitude at half-life (0.5N_f_), respectively. CG PA has the highest HR value among all the series, and the gap between HR and SR continues to decrease between $\varepsilon_{ta}$ 0.6–1.0%; however, when $\varepsilon_{ta}$ > 1.0%, the gap tends to increase again as the slope of SR decreases. CG OA is the only example that displays transition, and SR is higher than HR between $\varepsilon_{ta}$ 0.6–1.0%; as $\varepsilon_{ta}$ increases, a transition occurs at $\varepsilon_{ta}$ = 1.069% and HR is higher than SR at $\varepsilon_{ta}$ = 1.2%. The HR of UFG 1 and UFG 2 is 0, and because the SR of UFG 2 is approximately twice that of UFG 1 in the entire $\varepsilon_{ta}$, it can be confirmed that more rapid softening occurs in UFG 2. The HR for UFG 3, is the only positive HR among those of the UFGs and its value is larger than that of SR. Thus, hardening plays a dominant role, and the gap between HR and SR tends to decrease as $\varepsilon_{ta}$ increases.

#### 3.2.2. Hysteresis Loop

**Figure 4:**
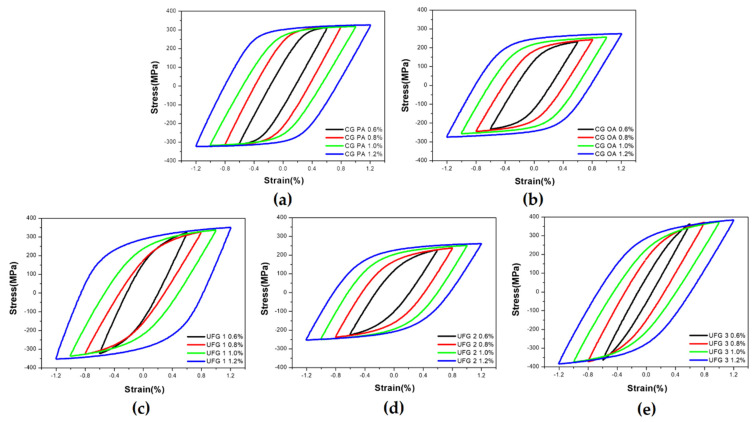
Evolution of hysteresis loops obtained from half-life of LCF tests: (**a**) CG PA; (**b**) CG OA; (**c**) UFG 1; (**d**) UFG 2; (**e**) UFG 3.

Figure 4 shows changes in the hysteresis loop for each series. Each hysteresis curve was obtained at 0.5N_f_. The shape of the hysteresis curve varies depending on aging conditions, SPD, and subsequent heat treatment process. The values of elastic strain amplitude ($\varepsilon_{ea}$) and $\varepsilon_{pa}$ can be confirmed by analyzing the shape of the hysteresis curve. As $\varepsilon_{pa}$ increases, the internal width of the hysteresis curve increases in all series, a trend that is in good agreement with that observed in earlier studies [52]. Generally, for a given $\varepsilon_{ta}$, high-strength materials tend to have a large $\varepsilon_{ea}$, and low-strength materials tend to have a large $\varepsilon_{pa}$. In the CG group, the inner width of the loop of CG PA is smaller than that of CG OA for the same $\varepsilon_{ta}$; this occurrence is due to the relatively high strength of CG PA (Figure 4a,b). The UFG group displays a similar tendency, with UFG 3, which has the highest strength, displaying the narrowest internal width over all $\varepsilon_{ta}$; UFG 2 displays a relatively wide internal width (Figure 4c–e).

**Figure 5:**
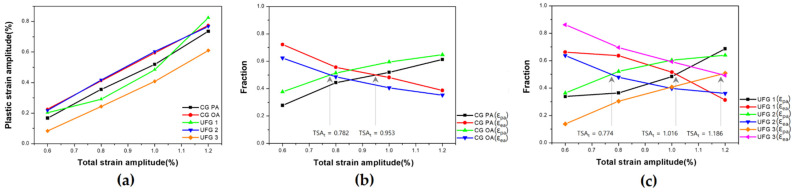
Relationship between total strain amplitude (*ε_ta_*) and plastic strain amplitude (*ε_pa_*) (**a**); Fraction of elastic-plastic strain amplitude (*ε_e,pa_*) with total strain amplitude (*ε_ta_*) (**b**,**c**), CG group, UFG group, respectively.

The change in $\varepsilon_{pa}$ as a function of $\varepsilon_{ta}$ and the results of the analysis of $\varepsilon_{ea}$ and $\varepsilon_{pa}$ fraction are shown in Figure 5. The tendency that $\varepsilon_{pa}$ increases with an increase in $\varepsilon_{ta}$ is consistent with that observed in previous research [52]. For UFG 1, the linearity decreases compared with that of the other series (Figure 5a), and this appears to be because the precipitates of UFG 1 arise due to under-aging. Thus, additional dynamic precipitation [18] can occur during LCF in a manner similar to that observed in previous research in which the LCF behavior changed notably due to dynamic precipitation in under-aged materials [22]. Interestingly, the $\varepsilon_{pa}$ of CG OA and UFG 2 materials containing OA precipitates are similar (Figure 5a); this phenomenon can be useful in understanding the LCF behavior of CG and UFG materials upon over-aging when exposed to similar $\varepsilon_{pa}$.

As $\varepsilon_{pa}$ increases in $\varepsilon_{ta}$, plastic deformation plays a critical role during cyclic deformation, therefore, it is important to analyze the fraction of $\varepsilon_{pa}$ and understand the behavior of a given plastic deformation. The fraction of $\varepsilon_{ea}$ and $\varepsilon_{pa}$ can be understood as the dependence of elastic and plastic deformation (EDD and PDD) on cyclic deformation and can be an indicator in identifying the dominant deformation mechanism in a specific $\varepsilon_{ta}$ for each series. The fractions of $\varepsilon_{ea}$ and $\varepsilon_{pa}$ were calculated for each $\varepsilon_{ta}$ and are shown in Figure 5b,c. In the CG group, CG OA displays a higher PDD than that of CG PA in the entire $\varepsilon_{ta}$ range, with transition occurring at $\varepsilon_{ta}$ = 0.782% (Figure 5b). In the UFG group, except for $\varepsilon_{ta}$ = 1.2%, the PDD is high and is in the order of UFG 2, UFG 1, and UFG 3 in the entire $\varepsilon_{ta}$ range (Figure 5b,c). UFG 3, which has a higher HR than SR, exhibits the lowest PDD in all the series; because the transition occurs at $\varepsilon_{ta}$ = 1.186%; plastic deformation is not dominant for the entire $\varepsilon_{ta}$ except for $\varepsilon_{ta}$ = 1.2% (Figure 5c).

#### 3.2.3. Microstructural Evolution after Cyclic Deformation

**Figure 6:**
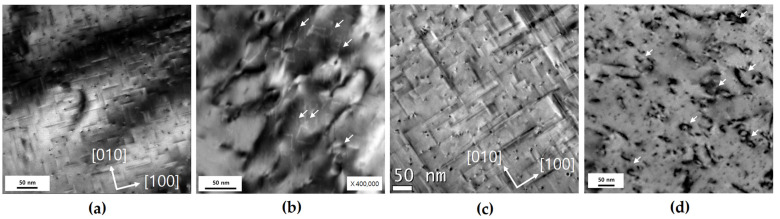
Transmission electron microscopy micrographs of characteristic microstructure of CG PA and CG OA before and after cyclic deformation: (**a**) and (**c**) precipitate morphology before LCF, β″, β, respectively; (**b**,**d**) evolution of microstructure after LCF at total strain amplitude ($\varepsilon_{ta}$) of 1.2%.

Figure 6 shows the TEM observation results of the morphology of the main strengthening precipitates β″ and β, which changes depending on the aging conditions and cyclic deformation of the CG material. The morphology, including the aspect ratio, observed in each material under peak-aging conditions and over-aging conditions matches well with the results obtained by other researchers for Al-Mg-Si alloys [22,53,54]. β″, the main strengthening precipitate, is sheared due to dislocation during LCF (Figure 6b) [53,54]. Thus, the precipitates that are reduced below the critical size are dissolved [40], and therefore, the contrast of the precipitates becomes less distinct compared with that before the test owing to the decrease in the strain field by β″ (Figure 6b) [49]. In terms of monotonic tensile properties, which are primarily concerned with strength and ductility, CG OA may be considered an inferior material to CG PA; however, during LCF, this comparison can be re-evaluated owing to the advantage posed by the slip behavior of the OA precipitates. Figure 6d shows that the CG OA tested at $\varepsilon_{ta}$ = 1.2% forms Orowan loops. Orowan loops are formed by the interaction between dislocations and non-shearable precipitates in CG OA; in addition, the homogeneity of materials that form Orowan loops when being deformed is acknowledged [22,43]. The formation of Orowan loops and loop patches as observed in the TEM images is in good agreement with the results obtained in previous research for OA Al-Mg-Si alloys [22,25].

**Figure 7:**
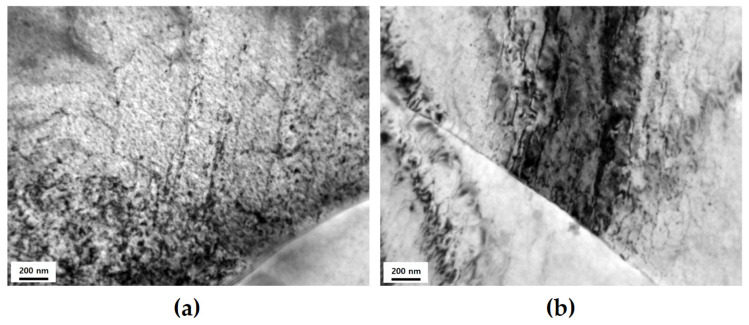
Transmission electron microscopy micrographs showing the dislocation substructures induced by cyclic deformation of CG PA after LCF: (**a**) slip bands at total strain amplitude ($\varepsilon_{ta}$) of 0.6%; (**b**) severely formed and localized slip bands at total strain amplitude ($\varepsilon_{ta}$) of 1.2%.

Shearing of the precipitates has a notable impact on LCF behavior and life. Shearable precipitates have a negative effect on the homogeneity of deformation after shearing [18,22,42], though it effectively hinders the movement of dislocation until shearing; however, after shearing, inhomogeneous deformation occurs owing to slip localization. Moreover, this effect can worsen as $\varepsilon_{ta}$ increases [55]. In Figure 7, the difference in slip band formation, during slip, with an increase in $\varepsilon_{ta}$ of CG PA is shown using TEM images. The formation of the slip band after $\varepsilon_{ta}$ = 1.2% intensifies and is localized compared with that at $\varepsilon_{ta}$ = 0.6% (Figure 7a,b); this intensified slip band accelerates localized deformation and further worsens the inhomogeneity of deformation [55].

**Figure 8:**
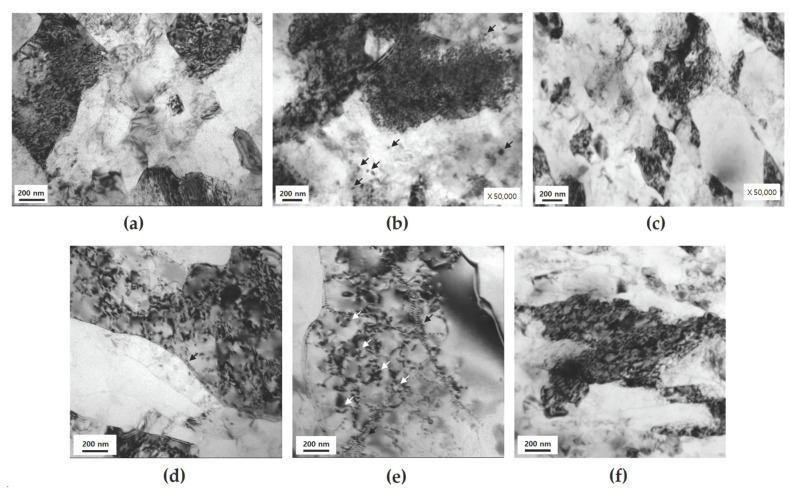
Transmission electron microscopy micrographs of characteristic microstructure of UFG group before and after cyclic deformation: (**a**–**c**) before LCF, UFG 1, UFG 2, and UFG 3, respectively; (**d**–**f**) microstructural evolution after LCF at total strain amplitude ($\varepsilon_{ta}$) of 1.2%, UFG 1, UFG 2, and UFG 3, respectively. The subgrain boundaries are marked by black arrows (**d**,**e**); Orowan loops and patches are indicated by white arrows (**e**).

**Figure 9:**
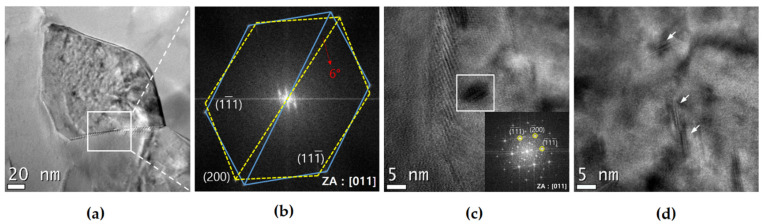
High resolution transmission electron microscopy micrographs of characteristic microstructure of UFG 3 before LCF: (**a**) nano-sized grain with diverse contrasts; (**b**) FFT SADP analysis for misorientation angle; (**c**,**d**) morphologies of very fine precipitates.

Figure 8 shows the TEM images of the microstructural changes undergone by the UFG group before and after LCF. For the UFG group, the most important factor in LCF behavior is the ability to resist softening, i.e., the stability of the microstructure toward deformation [28,46]. UFG 1 displays weak softening behavior during the LCF test, and after testing at $\varepsilon_{ta}$ = 1.2%, it was observed that the dislocation density of the grains decreases markedly and the formation of subgrain boundaries (Figure 8d). The reason for the weak softening is thought to be the formation of both very fine dynamic precipitates [18,22,56,57] with coherency [46] and subgrains that suppress the decrease in strength caused by the decrease in dislocation density. The TEM images of UFG 2 before testing indicate that a high density of dislocation exists, but the degree of tangled dislocations tends to decrease owing to the thermal process, and spherical and fragmented rod-like precipitates with sizes ranging between 30–80 nm are observed (Figure 8b). The morphology of the precipitates of SPD-treated materials can appear very different compared with that of conventional materials due to dynamic precipitation, the fragmentation of precipitates, and static precipitation [39,46,49]. Moreover, materials to which the PA conditions of CG materials and SPD are applied together are known to have incoherent precipitates [46]. After testing at $\varepsilon_{ta}$ = 1.2%, Orowan loops were observed inside the grain, which can be attributed to the non-shearable precipitates that formed during over-aging conditions (Figure 8e). The formation of Orowan loops can contribute to the homogeneity of deformation, but it is known that it can accelerate the annihilation of dislocations [52] and cause annihilation of the formed Orowan loops [43]. Thus, UFG 2 displays the features of microstructures that can increase dynamic recovery, i.e., low microstructural stability. Similar to UFG 1, the formation of subgrains may be a factor in resisting softening, but the above-mentioned softening factors are thought to be more dominant. The detrimental reason concerning the low microstructural stability of UFGs appears to be that the rate of microstructural change is rapid and inhomogeneous. Upon observation of the microstructure of UFG 1 and UFG 2, it was noted that the recovery rate due to dynamic recovery is different for each area of the microstructure. Furthermore, when the microstructure change rate is fast (UFG 2) compared to when it is slow (UFG 1), the rate of inhomogeneous microstructure change per cycle is more severe. That is, the UFG 2, which has an LCF life of approximately 41% compared with that of UFG 1 at $\varepsilon_{ta}$ = 1.2%, undergoes approximately 2.133-times greater inhomogeneous dynamic recovery within that time. UFG 2 may have a relatively low driving force for recovery due to heat treatment after SPD, and although it exhibited a lower PDD compared with that of UFG 1 at $\varepsilon_{ta}$ = 1.2%, it displayed a greater softening. Hence, the microstructural stability toward dynamic recovery in LCF stems from suppressing the movement of dislocations. UFG 3 is very different from UFG 1 and UFG 2. No significant difference in the microstructure can be observed before and after LCF deformation at $\varepsilon_{ta}$ = 1.2%, and there is still a high dislocation density inside the grains (Figure 8f). Accordingly, UFG 3 has the highest microstructural stability at $\varepsilon_{ta}$ = 1.2%. Figure 9 shows the results obtained from high resolution TEM of the microstructural characteristics of UFG 3 before LCF. Upon observation of some grain boundaries, a contrast that appears to be a subgrain boundary was noted (Figure 9a). Fast Fourier transform (FFT) selected area diffraction pattern (SADP) analysis confirms that it is a low angle grain boundary (LAGB) with a 6 ° difference in orientation angle (Figure 9b), and this is in good agreement with the observations of previous studies on LAGB found in UFG materials [46]. Inside the grain, the contrasts formed from dislocation and precipitates are visible (Figure 9a), and there are precipitates that are 5 nm in size (Figure 9c); furthermore, needle-shaped precipitates ranging in size between 5–7 nm (Figure 9d) are present. The needle-shaped precipitates are finer than the 30–60 nm-sized needle type β″ found in CG PA; however, a previous study on low-temperature heat treatment after SPD conducted on Al-Mg-Si alloys found that the corresponding fine precipitates were β″ [39]. Spherical precipitates form because when precipitation occurs in a microstructure with a high dislocation density, atomic diffusion is enhanced by pipe diffusion near randomly distributed dislocations, resulting in isotropy [39]. Other studies on the Al-Mg-Si series discovered that these fine precipitates after SPD are β″ [49] and are formed by precipitate fragmentation due to severe shear deformation that occurs during SPD; the precipitates are effective in dislocation pinning [49] and can have various morphologies and coherencies [46]. Therefore, for UFG 3, there are two reasons why it did not show continuous softening behavior, (i) when a very fine precipitate with coherency interacts with a dislocation, dynamic recovery is suppressed by effectively reducing the movement of the dislocation, and (ii) exposure to relatively low $\varepsilon_{pa}$ due to microstructural characteristics.

#### 3.2.4. Low Cycle Fatigue Life Using Strain-Based Approach

The LCF life of a material is related to $\varepsilon_{ta}$, and the Basquin and Coffin–Manson relationships are mainly used to determine the $\varepsilon_{ea}$ and $\varepsilon_{pa}$ versus LCF life, respectively [28,47]. The relationship between $\varepsilon_{ta}$ and LCF life is expressed using the equation below [58].

(3) $$\varepsilon_{ta}=\sigma \prime_{f}^{\;}{\left(E\right)}^{-1}{\left(2N_{f}\right)}^{b}+\varepsilon \prime_{f}^{\;}{\left(2N_{f}\right)}^{c},\;$$

where *E*, $\sigma \prime_{f}^{\;}$, and $\varepsilon \prime_{f}^{\;}$ are the Young’s modulus, fatigue strength coefficient, and fatigue ductility coefficient, respectively; *b* and *c* are the fatigue strength exponent and fatigue ductility exponent, respectively.

**Figure 10:**
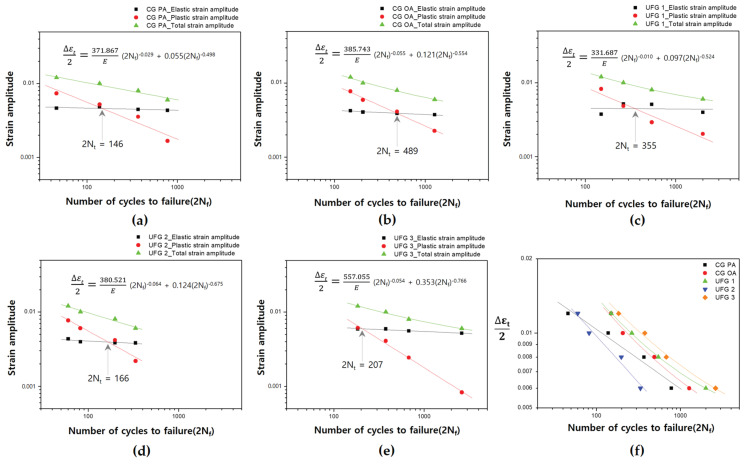
Basquin and Coffin–Manson type plots under various strain amplitude conditions: (**a**) CG PA; (**b**) CG OA; (**c**) UFG 1; (**d**) UFG 2; (**e**) UFG 3; (**f**) CG, UFG groups.

Figure 10 shows the relationship between $\varepsilon_{ea}$, $\varepsilon_{pa}$, and $\varepsilon_{ta}$ and the LCF life for each series. As the test was conducted under $\varepsilon_{ta}$ control, the values required for plotting and calculation were obtained from the 0.5N_f_ conditions. Plastic deformation behavior is very important in LCF, and the equation which shows the relationship between $\varepsilon_{pa}$ and stress amplitude ($\sigma_{a}$) helps in understanding the importance thereof [20,22].

(4) $$\sigma_{a}=k^{\prime }{\left(\varepsilon_{pa}\right)}^{n^{\prime }},\;$$

where *k′* and *n′* are the cyclic strength coefficient and cyclic strain hardening exponent, respectively.

The estimated LCF parameters based on Equations (3) and (4) are presented in Table 3. The LCF life is a value determined by combining each exponent and coefficient, and $\sigma \prime_{f}^{\;}$ and $\varepsilon \prime_{f}^{\;}$ may be related to the monotonic tensile property. UFG 3 had the highest values of $\sigma \prime_{f}^{\;}$, $\varepsilon \prime_{f}^{\;}$, and *k′* among all the series. However, a recent LCF paper on Al-Mg-Si alloys reported that the LCF parameters used in the above equations can display various trends and value ranges depending on aging and process conditions [22].

*n′* is a parameter that indicates the ease of dislocation movement during cyclic deformation [59] and has high values in CG OA and UFG 2 which contain microstructures that facilitate dislocation movement. By contrast, CG PA and UFG 3, which are relatively difficult to dislocation glide and cross-slip, have low values of *n′*, which are in good agreement with that obtained in previous studies [20,22,59]. Within the UFG group, *n′* increased in the order of UFG 3, UFG 1, and UFG 2 which have high microstructural instability. Thus, *n′* can be used as a valid parameter to indicate the dynamic recovery due to dislocation rearrangement of UFG materials in Al-Mg-Si alloys, and additional studies will help to clearly establish this relationship.

In the UFG group, the faster softening progresses, the shorter the LCF life; a trend that agrees with that observed in earlier research [27,28,47]. For UFG 3, in which continuous softening did not occur, the longest LCF life was observed over the entire $\varepsilon_{ta}$ range (Figure 10f). By contrast, UFG 2, in which the most rapid and severe softening occurred, exhibited the shortest LCF life of all the series, thereby indicating the possibility that softening can be used as a useful parameter to predict the LCF life of UFG materials in Al-Mg-Si alloys.

In general, from a macroscopic viewpoint, materials with high ductility may exhibit long LCF life under high $\varepsilon_{ta}$ conditions, while materials with high strength may exhibit long LCF life under low $\varepsilon_{ta}$ conditions [47,55]. For the CG group used in this study, the ductility is similar to or lower than those of most of the UFG group except for UFG 2; thus, there is no advantage in displaying a long LCF life at high $\varepsilon_{ta}$. CG OA exhibited a shorter LCF life compared with that of the UFG group except for UFG 2; moreover, softening suppression due to the microstructural stability of some UFG materials was also a contributing factor to the improvement in LCF life. However, despite its superior ductility compared with that of the UFG group, CG PA had a very short LCF life compared with all the examples investigated, suggesting that monotonic properties may not match cyclic properties in Al-Mg-Si alloys. For the Al-Mg-Si alloys of other studies formed under the PA condition, the LCF life was shorter than that of the OA condition in the $\varepsilon_{ta}$ range of this study [20,22]. As previously mentioned, the reason CG PA displays inferior LCF life is due to intensified strain localization caused by the interaction of dislocation with shearable precipitates. For monotonic straining in which dislocations do not reciprocate, the effect on slip and related properties that are caused by interaction with coherent precipitates is relatively small [46]; however, for cyclic straining with repetitive movements, it has a very large impact through locally concentrated slip and persistent slip bands (PSB) [22] that cause early failure [46]. By contrast, CG OA was inferior to CG PA in terms of all tensile properties, but because of the advantage of homogeneous deformation attained from interacting with non-shearable precipitates, it exhibits a superior LCF life compared with that of CG PA over the entire $\varepsilon_{ta}$ range. Interestingly, in the high $\varepsilon_{ta}$ range, the difference in LCF life between CG OA and UFG 1 and 2 decreases and becomes more marked for UFG 2 and CG PA, thereby leading to a transition (Figure 10f). The precipitates of CG OA and UFG 2 are in the OA condition and both examples have non-shearable precipitates. Here, a decrease in slip irreversibility [43] may occur at high $\varepsilon_{ta}$, resulting in an increase in slip planarity [28,60]. Recent studies [28,60] show that the LCF life increases with increasing slip planarity; thus, the advantage of homogeneous fine wavy slip [61] caused by the non-shearable precipitates of Al-Mg-Si alloys is combined with an increase in slip planarity; hence, LCF life can be improved at high $\varepsilon_{ta}$.

## 4. Conclusions

A systematic discussion was conducted in terms of the effect of microstructural evolution and strengthening mechanisms on the cyclic deformation behavior and LCF life of CG and UFG 6005 Al alloys under various aging conditions. The results may be summarized as follows:

1. The state of precipitates under different aging conditions significantly influenced the cyclic stress response of both CG and UFG Al-Mg-Si alloys. The CG PA alloy displayed initial cyclic hardening followed by saturation, exhibiting the highest HR value compared to other series. The CG OA alloy showed cyclic softening behavior nearing saturation, with the transition occurring at $\varepsilon_{ta}$ = 1.069% for HR and SR values. In contrast, the UFG materials demonstrated continuous cyclic softening, except for UFG 3, where the HR was the only one among the UFGs to be positive, and its value surpassed that of the SR.
2. The CG group displayed different tendencies between monotonic tensile properties and LCF properties depending on the state of the precipitates. CG PA with shearable precipitates exhibited strain localization and inhomogeneous deformation, and this tendency intensified as $\varepsilon_{ta}$ increased, resulting in a shorter LCF life over the entire $\varepsilon_{ta}$ range than that of CG OA with non-shearable precipitates, thereby enhancing deformation homogeneity.
3. In the UFG group, LCF life decreased with the degree of cyclic softening. Softening is caused by dynamic recovery during cyclic deformation, and materials with an inherent ability to suppress the rearrangement of dislocations displayed high microstructural stability. The stability of a microstructure is closely related to the state of the precipitates, and UFG 2, which had coarse and incoherent precipitates, exhibited the most severe softening and shortest LCF life. By contrast, UFG 3, which had very fine and coherent precipitates and had the lowest $\varepsilon_{pa}$ due to microstructural characteristics, did not exhibit continuous softening and showed the longest LCF life. Additionally, UFG 3 had the highest values of $\sigma \prime_{f}^{\;}$, $\varepsilon \prime_{f}^{\;}$, and *k′* among all the series. Cyclic strain-hardening exponent *n′*, one of the strain-based LCF parameters, showed an increasing nature as the dynamic recovery of UFGs became more severe.

## Figures and Tables

**Table 1:** Chemical composition (wt.%) of the 6005 Al alloy.

Si	Fe	Cu	Mn	Mg	Cr	Zn	Ti	Al
0.9	0.13	0.008	0.008	0.52	0.01	0.005	0.005	Bal.

**Table 2:** Monotonic tensile properties of the 6005 Al alloy.

Materials	σ_YS_ (MPa)	σ_UTS_ (MPa)	El (%)	TS/YS	TT (MJm^−3^)
CG PA	301.6	326.3	16.7	1.082	51.716
CG OA	259.5	284.6	9.5	1.097	25.129
UFG 1	350.4	374.1	10.1	1.067	35.302
UFG 2	271.2	289.9	4.4	1.069	11.421
UFG 3	355.7	388.0	12.6	1.091	45.636
6063 PA(CG) [22]	264.3	285.7	17.6	1.08	-
6061 PA(CG) [20]	300	338	13	1.13	-

Note: σ_YS_ = yield strength; σ_UTS_ = ultimate tensile strength; El = total elongation; TT = tensile toughness.

**Table 3:** Strain-based various low cycle fatigue parameters (CG, UFG groups).

Parameters			Materials			
Description	Symbol	CG PA	CG OA	UFG 1	UFG 2	UFG 3
Fatigue strength coefficient (MPa)	$\sigma \prime_{f}^{\;}$	371.867	385.743	331.687	380.521	557.055
Fatigue strength exponent	*b*	−0.029	−0.055	−0.010	−0.064	−0.054
Fatigue ductility coefficient	$\varepsilon \prime_{f}^{\;}$	0.055	0.121	0.097	0.124	0.353
Fatigue ductility exponent	*c*	−0.498	−0.554	−0.524	−0.675	−0.766
Cyclic stress coefficient (MPa)	*k′*	326.911	279.164	353.387	265.644	385.319
Cyclic strain hardening exponent	*n* *′*	0.025	0.134	0.062	0.108	0.026

## Data Availability

The datasets generated during and/or analyzed during the current study are available from the corresponding author on reasonable request.
